# The nicotinamide hypothesis revisited—plant defense signaling integrating PARP, nicotinamide, nicotinic acid, epigenetics, and glutathione

**DOI:** 10.1002/1873-3468.70146

**Published:** 2025-08-20

**Authors:** Torkel Berglund, Anna B. Ohlsson

**Affiliations:** ^1^ Department of Industrial Biotechnology School of Engineering Sciences in Chemistry, Biotechnology and Health, KTH Royal Institute of Technology Stockholm Sweden

**Keywords:** defense, DNA hypomethylation, epigenetics, glutathione, histone acetylation, nicotinamide, nicotinic acid, poly(ADP‐ribose) polymerase, reactive oxygen species, stress signaling

## Abstract

A previously presented hypothesis, here updated, proposed nicotinamide produced by the activity of poly(ADP‐ribose) polymerase (PARP) as a stress signaling compound in plants. PARP activation by DNA strand breaks caused by oxidative stress leads to the metabolization of NAD to ADP‐ribose units and nicotinamide. Also, NAD‐degrading histone deacetylases (sirtuins) produce nicotinamide. We suggest that nicotinamide, either alone or acting through its metabolite nicotinic acid, will generate epigenetic changes in the plant. We propose that this is an early step in a general stress response, making the DNA more accessible to the transcription machinery and specific stress signaling substances to activate the defense genes needed for the present stress situation. The role of glutathione in this context is discussed.

## Graphical abstract

Nicotinamide (NIC) and nicotinic acid (NIA) are proposed as stress signaling compounds in plants. Oxidative stress may lead to single strand breaks (SSB) in DNA, which activate poly(ADP‐ribose) polymerase (PARP). NIC and NIA are then formed from NAD. NIC and NIA can promote epigenetic changes leading to the expression of defense genes specific for the present stress situation.

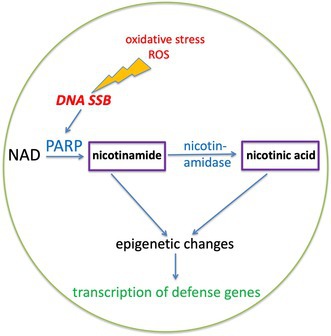

## Abbreviations

3‐AB 3‐aminobenzamide

AAPH 2,2′‐azobis(2‐amidinopropane) dihydrochloride

ABA abscisic acid

cADPR cADP‐ribose

GSH glutathione

IAA indole acetic acid

JA jasmonic acid

MeJA methyl jasmonate

NAD nicotinamide adenine dinucleotide

NIA nicotinic acid

NIC nicotinamide

PAL phenylalanine ammonia‐lyase

PARP poly(ADP‐ribose) polymerase

ROS reactive oxygen species

SA salicylic acid

TRIG trigonelline (N‐methylnicotinic acid)

UV‐B ultraviolet‐B‐radiation

Plants are of paramount importance for life on Earth, including human life. Accordingly, it is important to understand how plants function and respond to their environment on short and long timescales. On a short timescale (hours, days), plants must handle rapid changes in their environment, such as biotic stress, which demands fast and pronounced changes in gene expression. In this extended hypothesis, we highlight some effects of nicotinamide (NIC) and nicotinic acid (NIA) in plants, such as DNA hypomethylation and elevated defense gene expression. We underline our proposal that NIC and NIA are only a couple of many stress signaling molecules; their role may be as openers of DNA for interaction with other stress signaling molecules that fine‐tune the responses. During periods of limited threat, growth is favored along with basic defense against drought or cold and other kinds of abiotic stress, experienced regularly by most plants. Both growth and defense are energy‐consuming processes. Thus, to cope with limited energy resources, in a broad manner, plants sometimes have to selectively prioritize either growth or defense [1, 2]. In a rapidly changing environment, genetic resources have to be used efficiently by regulating gene expression. Mechanisms that can control gene expression in response to stimuli may be inherited during cell division and may even persist over generation borders [3, 4]. It is increasingly clear that epigenetics has an important role in such mechanisms [3, 5].

Epigenetics is considered one important future plant biotechnology track [6, 7]. From an applied point of view, the impact of epigenetic mechanisms as a basis for selection of high yield crops and stress tolerance has been demonstrated regarding biotic as well as abiotic stress [5, 8, 9, 10].

Most types of stress in plants are associated with increased cellular levels of reactive oxygen species (ROS) [11, 12], which can cause an increase in DNA strand breaks, for example, single strand breaks, as well as many other types of damage. This is a potential threat to the cells that demands an immediate defensive reaction; DNA, the core of life, must be protected. Since many different types of oxidative stress can cause DNA damage, there should logically be a general initial response reacting to as many potential causes of the ROS increase and DNA damage as possible. Such a response may have roots in ancient biology when life forms first interacted with oxygen, a persistent molecular threat to vital biomolecules. Speculatively, other more specialized defense responses were built upon this basic self‐protective fundament.

A role for NIC in DNA damage and repair in animal cells has been highlighted [13, 14, 15]. But information concerning the role of NIC as well as its metabolite NIA in plants is scarce. Thirty years ago, we presented a hypothesis suggesting NIC as a signal mediating compound in association with oxidative stress and poly(ADP‐ribose) polymerase (PARP) activity in eukaryotic cells, especially plants [16, 17]. Here, we highlight some further data in support of this hypothesis and extend it toward connections between epigenetic effects and defense, where PARP, NIC, and NIA play important roles.

NIC is the active part of the cofactor nicotinamide adenine dinucleotide (NAD), which is essential within energy metabolism, such as in glycolysis, the citric acid cycle, mitochondrial respiration, and various signaling pathways [18, 19]. The importance of NAD for growth and survival in animal cells can be exemplified by inhibition of NAD biosynthesis to counteract inflammation and the growth of cancer cells [20]. One example of the importance of NAD in plants is the phosphorylation of NAD to 3'‐NADP by the *Xanthomonas* avirulence factor AvrRxo1, interfering with the growth of the plant [21].

NIC can be released from NAD by activity of PARP [22] or other as yet unidentified ADP‐ribosylating activities [23], via a reaction that forms cyclic ADP‐ribose (cADPR) [24], and by NAD‐dependent and NAD‐degrading histone deacetylases (sirtuins) [25]. We do not know how much NIC is released from these reactions, although the reactions as such are of great importance for the plants survival. The activity of sirtuins connects NIC to gene expression, since histone acetylation is of great importance for chromatin structure and the expression of genes [26]. NIC is a product and inhibitor of both PARP [22] and sirtuin [25] activities. Addition of exogenous NIC to cultured plant cells or intact plants has in two ways a positive impact on the NAD pool. First, NIC can increase the level of NAD by inhibiting PARP‐mediated NAD degradation. This is important for plant growth [27]. Second, in plants NIC is metabolized to NIA, which in turn is a precursor for the NAD salvage pathway generating NAD [28]. Furthermore, exogenous application of NIC can induce a plethora of general defensive activities and decreased level of DNA methylation [16, 17, 29, 30, 31]. The related molecule NIA induces the same kind of defense response [29, 30]. In addition, transcriptomic analysis of roots from spruce seedlings grown from NIC‐treated seeds has revealed differentially expressed genes connected to defense and DNA methylation [32]. Altogether, this points to NIC and NIA being general defense regulators, enabling more specific gene expression and defensive activities via various regulatory molecules and mechanisms.

In this hypothesis paper, we have concentrated the discussion on the reactions and metabolism closest to our formulated hypothesis, even though there are, of course, a lot of other players in the cell that have an impact on the same processes.

### Hypothesis

In this paper, our former hypothesis regarding the role of NIC in stress response in plants [17] is considered and extended in the light of new discoveries. We suggest that in response to most types of stress that increase PARP activity, leading to NAD metabolization to NIC, considerable amounts of the plant cell's chromatin will be opened by epigenetic changes, giving access to the transcription machinery for a certain period of time. In other words, there is a transient emergency standby system to enable upregulated transcription. Broad gene expression is thereby made possible, but it is likely that only a specific subset of genes is in fact activated, depending on which transcription promoting factors are present. We hypothesize that NIC and NIA can act as signal mediating compounds promoting this open state of transcription via epigenetic mechanisms such as changes in histone modification and/or DNA methylation.

**Fig. 1:**
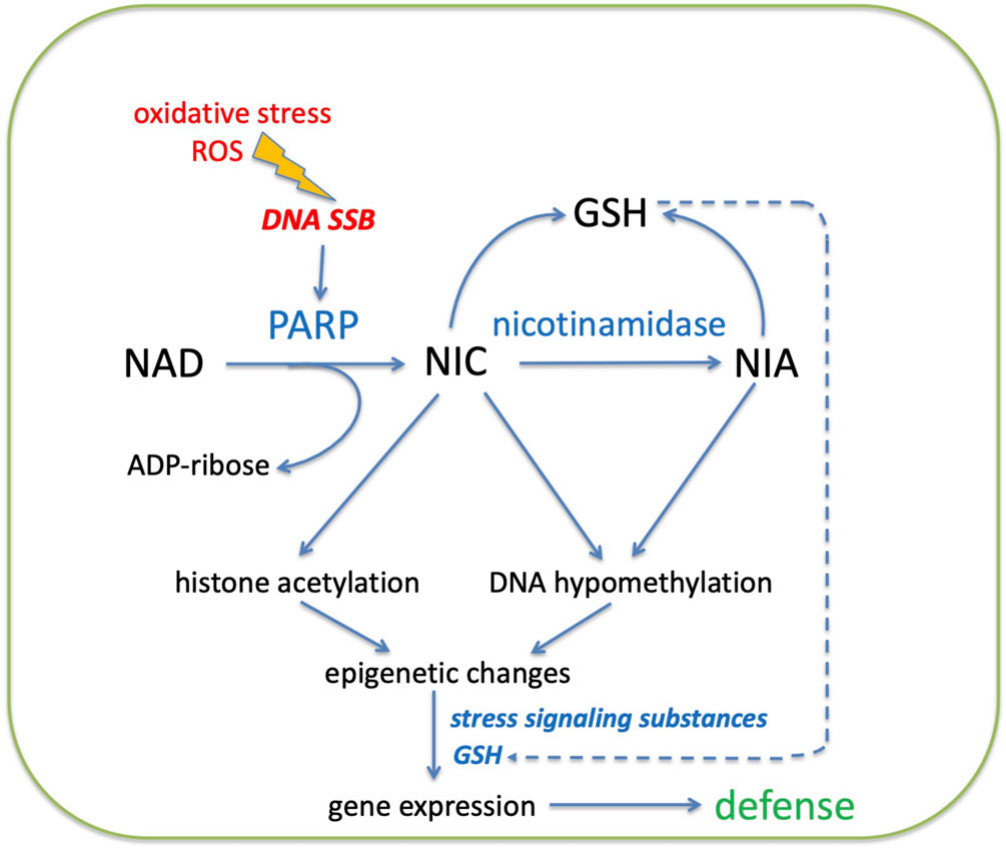
Illustration of the nicotinamide hypothesis. When single strand breaks (SSB) are formed in DNA, caused by for instance reactive oxygen species (ROS), poly(ADP‐ribose) polymerase (PARP) is activated. This activity will metabolize NAD to ADP‐ribose and nicotinamide (NIC). Nicotinic acid (NIA) is subsequently formed from NIC by nicotinamidase. Both NIC and NIA give rise to DNA hypomethylation. NIC may also increase the acetylation of histones by inhibition of histone deacetylase activity. Epigenetic changes like decreased DNA methylation as well as increased histone acetylation promote a more open and available chromatin, giving access to appropriate stress signals for transcription of defense genes. NIC and NIA will also increase the level of glutathione (GSH), which may promote the sensitivity to defense signaling substances like salicylic acid and jasmonic acid.

The initial release of NIC from NAD via PARP activity, as well as the metabolization of NIC to NIA, is of central importance for our expanded hypothesis, illustrated in Fig. 1. We suggest that PARP activity, induced by DNA strand breaks via, for example, ROS or other DNA nicking processes, leads to NIC release from NAD and NIC transformation to NIA. Then, NIC and NIA generate an epigenetic effect, promoting the availability of DNA for transcription. NIC release from NAD via cADPR formation is also considered. Furthermore, we regard a NIC and NIA induced increase in glutathione (GSH) level as potentially important in this context by promoting the sensitivity to defense signaling substances such as salicylic acid and jasmonic acid.

New information from our own work and results from available literature support our original hypothesis that NIC and its metabolites are defense‐promoting compounds, and that epigenetic effects and GSH metabolism have important functions in their action. A thorough understanding of the underlying mechanisms may provide us with powerful tools to influence plant performance in forestry and agriculture. We suggest that NIC and NIA, also known as vitamin B_3_, as well as agents influencing the biosynthesis and metabolism of these compounds are such tools.

## Discussion

### Poly(ADP‐ribose) polymerase (PARP), a starting point

Poly(ADP‐ribose) polymerases (EC 2.4.2.30) are known to influence a broad spectrum of physiological processes in animals and plants, such as DNA repair and regulation of gene activity [22, 24, 33, 34]. Enzymes with PARP function are found in the PARP superfamily, which also includes PARP‐like proteins lacking functional PARP activity [35, 36]. PARP activity is increased as a response to DNA damage caused by, for example, oxidative stress [37, 38, 39]. As a result, NAD is metabolized to NIC and ADP‐ribose, whereby ADP‐ribose units are used to create ADP‐ribose polymers on various proteins, influencing their activity in the nucleus as well as other compartments [40].

From animal cell studies, it is known that PARP activity leading to increased poly(ADP‐ribosyl)ation can decrease DNA methylation by inhibiting DNA methyltransferase 1 (DNMT1), and that PARP inhibition by 3‐aminobenzamide (3‐AB) can increase DNA methylation, for example, in CG‐rich islands [41, 42]. In this way, chromatin packing can be changed, which in turn influences the availability of genes for transcription. The importance of poly(ADP‐ribosyl)ation in plant defense was demonstrated by Briggs et al. [43]. The role of the second product of the NAD‐cleaving process, NIC, is generally not considered in the literature except as a PARP or sirtuin inhibitor or as a precursor for the NAD salvage pathway. We suggest that NIC produced by PARP also has important functions in stress signaling.

### PARP and defense

As mentioned, PARP has a very important metabolic position in plants [22]. A reduction in PARP activity improves plant cell division and increases the fresh weight of plants under nonstress conditions, which may be explained by low NAD degradation, which is positive for energy metabolism [27]. On the other hand, as we know that growth stalls during stress response conditions, it appears logical that in stress situations and high PARP activity, the message would be signaling a decrease in cell division and DNA replication to counteract the establishment of mutations.

Research data point at an important role for PARP in plant defense on top of its function in DNA repair and ADP‐ribosylation, for example, in promoting pathogen defense [43] and response to microbial attack in the form of callose and lignin deposition, pigment accumulation, and phenylalanine ammonia lyase (PAL) activity [44].

This in turn suggests a potential role for NIC and its metabolite NIA in basic defense initiation and regulation. NIC is a natural PARP inhibitor [22]. If this were its main or sole function, one would primarily expect the effects of NIC addition to plants to be similar to those reported in association with PARP downregulation, such as enhanced growth [27, 45]. However, when plants are exogenously exposed to NIC, there are several other responses. Of principal interest is the onset of a broad defense potentiation. Despite the importance of poly(ADP‐ribosyl)ation for defense regulation in plants [34], we affirm that the release of NIC as a signaling molecule is itself significant with respect to PARP activity.

Connections between PARP and defense in *Arabidopsis* plants has been studied using transgenic plants. Downregulation of PARP1 and PARP2 reduced the defense against pathogens [34], which could possibly depend on reduced level of NIC and thereby absent stress signaling. In another study using PARP2 downregulated plants, abscisic acid (ABA) responsive genes and related defense increased, suggested to depend on cADP‐ribose formation from NAD, a reaction also forming NIC [46]. Downregulation of PARP has also been studied using chemical inhibitors such as 3‐methoxybenzamide (3‐MB) and 3‐AB [43, 44, 45], resulting in decreased defense parameters. The lower NIC level due to lowered PARP activity may be an explanation for the weakened defense. We cannot assess the importance of NIC in these examples. More research is needed, but to consider changed levels of NIC is an alternative way of interpreting these data from the literature, where the focus has primarily been on NAD levels, PARP activity per se, ADP‐ribose, and growth.

PAL is a gateway enzyme connecting primary metabolism and the phenylpropanoid/flavonoid pathway, the latter of which is of considerable importance for biotic defense [47]. The synthetic PARP inhibitor 3‐AB caused a decrease [48] and NIC addition caused an increase [49] in PAL activity in *Catharanthus roseus* cell cultures. Anthocyanin biosynthesis is part of the phenylpropanoid/flavonoid pathway, and decreased PARP activity resulted in decreased anthocyanin levels and decreased transcription of genes encoding enzymes involved in anthocyanin biosynthesis [45]. This is in line with results showing that NIC addition to *C. roseus* cell culture increased the content of anthocyanins [50] and increased the expression of *chs2* in *Pisum sativum* plants encoding chalcone synthase (CHS) [51], which is a strategic enzyme within the biosynthetic pathway leading to anthocyanins.

Thus, from our new perspective, a low PARP activity is a basic cellular state with low NIC signal generation. On the other hand, in association with PARP‐activating stress, a defense‐promoting NIC‐based signal chain is established, including nicotinamidase activity, metabolizing NIC to NIA. In this situation, nicotinamidase has two main functions. First, it disarms NIC as a feedback inhibitor of PARP (and NAD‐dependent sirtuins), and second, it creates a new signal mediating molecule, NIA, which is not a PARP inhibitor [52].

Furthermore, there is a possibility that NIC can also influence the activity of PARP‐like proteins such as RCD1 and SRO, which are involved in stress responses, and in this way influence a broad array of defense‐associated activities [24].

Increased levels of the growth factor ABA and abiotic stress resistance were demonstrated in PARP2‐deficient *Arabidopsis* plants [46]. As suggested by the authors, this could be due to an increased NAD level, steering NAD metabolism toward cADPR, which in turn leads to Ca^2+^ mobilization and ABA signaling [24]. It is noteworthy that in the formation of cADPR from NAD, NIC (and probably NIA) is alsoproduced.

### Nicotinamide has DNA‐hypomethylating and defense‐activating effects in plants

As speculated [17], NIC could be a link between oxidative stress, PARP activity, DNA methylation, and defense response in plants. A DNA hypomethylating effect of PARP activity [41, 42], but also of NIC [53], has been observed in animal cells. This is in line with results showing that the NAD metabolite NIC, a product of PARP activity, is a DNA hypomethylating compound in intact plants as well as in plant cell cultures. Nicotinamide treatment caused a decreased level of DNA methylation in needles of 15‐week‐old spruce seedlings grown from seeds soaked in NIC for a couple of hours [31] and in *P. sativum* shoot culture exposed to NIC in the culture medium [29]. Furthermore, in roots of 3‐month‐old spruce seedlings treated with NIC at seed stage, approximately 350 genes involved in epigenetic regulation and stress response were differentially expressed, among them DDM1 (known to promote DNA methylation), which was downregulated, and MYB77 and chitinases (stress response genes), which were upregulated [32]. Apparently, the seed treatment with NIC may have established an epigenetic state, persisting through many cell divisions during growth of the plant.

It has been shown that ultraviolet‐B radiation (UV‐B) causes elevated NIC levels and activation of defense [54] as well as DNA hypomethylation [55, 56]. Furthermore, it is known that UV‐B exposure of plants reduces the expression of DDM1 [57] and that the loss of DDM1 can enhance pathogen resistance in *Arabidopsis* [58]. As mentioned, NIC treatment can cause a downregulation of DDM1 expression and upregulation of stress response genes [32]. Speculatively, UV‐B exposure could via NIC formation result in the abovementioned effects. However, it is hard to be certain whether NIC or NIA caused the downregulation of DDM1, since exogenously added NIC may be metabolized to NIA. One way to elucidate this may be to inhibit nicotinamidase by, for example, nicotinaldehyde [59] or by using nicotinamidase mutants [28].

### Nicotinamide, nicotinic acid and priming

Plant priming is a broad concept used to improve plant tolerance [60, 61, 62]. By treatment of the plant with a mild stress or certain elicitor compounds, stress signaling will occur, and the plant will respond faster and stronger in later stress situations, for instance, by synthesis of stress signaling substances/growth factors, such as jasmonic acid (JA) [63] and salicylic acid (SA) [64].

There is an overlap between effects associated with priming on one hand, and effects of NIC/NIA on the other hand, concerning, for example, epigenetics and the redox state in the cell. A hallmark of priming is the generation of ROS and oxidative stress [61], in turn associated with DNA‐strand breaks, which trigger PARP activity [37, 38, 39]. This leads to the degradation of NAD into NIC, which in plants is further metabolized to NIA. NIC and NIA can counteract oxidative stress by increasing the GSH level [29], which may be decisive for the sensitivity of the stress response to, for example, JA and SA.

Epigenetic mechanisms are known to be involved in the priming process, even though many questions regarding the mechanisms behind priming remain unanswered [61, 65]. As discussed, epigenetic effects of NIC have also been shown; a decreasing effect on DNA methylation [29, 31], downregulation of the DNA methylation‐promoting gene DDM1 [32] and an increasing effect on sirtuin acetylation [26, 66].

We suggest that NIC and NIA may be formed during the priming process and that they can constitute an aspect of plant priming.

One plant protection strategy in which priming is suggested to be involved is the use of somatic embryogenesis (SE) for the production of spruce plants tolerant against pine weevil attack [67, 68]. An even higher degree of protection was achieved when the SE plants were sprayed with methyl jasmonate (MeJA) [68]. The preparation of SE plants is known to involve oxidative stress [69]. Indeed, it is known that oxidative stress can result in DNA hypomethylation and other epigenetic changes [70] and that epigenetic changes are involved in priming [61]. In line with this, it has been shown that DNA hypomethylation is important for SE development [71]. One possible interpretation is that in the SE process oxidative stress would result in NIC formation via PARP, leading to epigenetic changes making the chromatin more inviting to stress signal substances, such as MeJA.

### Inhibition of sirtuin activity by NIC may promote histone acetylation and defense metabolism

Sirtuins are enzymes occurring in different phyla from archaea to humans, with important regulatory functions involved in, for example, metabolism, chromatin silencing, and genomic stability [26, 72]. They were first discovered in yeast and were named SIR2 (silent information regulator2) [73]. Similar proteins in mammals are named SIRT1‐7 and in plants SRT [74]. One sirtuin class in plants is NAD‐dependent protein deacetylases, which consume NAD and release NIC during the deacetylation process. *Arabidopsis* contains two sirtuins, and so does rice [26]. Furthermore, it has been demonstrated that the rice sirtuin OsSRT1, in addition to deacetylase activity, also shows a mono‐ADP‐ribosylation activity [75]. In both of these activities, NIC will be released, and the authors discuss a link between the mono‐ADP ribosylation and increased tolerance to stress.

NIC [25, 26, 66] is a natural inhibitor of sirtuins. Sirtuin inhibition results in histone hyperacetylation, leading to a more open chromatin and accessible DNA, which in turn promotes transcription of genes taking part in, for example, defense and senescence [26].

The histone deacetylase HDA6 plays important roles in gene silencing [76]. HDA6 and the DNA methylation promoting DNA methyltransferase1 (MET1) seem to be dependent on each other and act together, promoting histone deacetylation and DNA methylation leading to gene silencing [77, 78]. Inhibition of either or both enzymes would result in a more open chromatin. Considering our findings that NIC treatment of plants can result in DNA hypomethylation [29, 31], and that NIC is a sirtuin inhibitor [25, 26, 66] we suggest sirtuin inhibition as one point where NIC can have an epigenetic effect through increased histone acetylation as well as decreased DNA methylation.

### Oxidative stress and glutathione

Oxidative stress can cause a decrease in global DNA methylation in plants [70]. ROS‐generating treatments, including the free radical generating compound 2,2′‐azobis(2‐amidinopropane) dihydrochloride (AAPH) [29], the herbicide paraquat [79], or the naphthoquinone juglone [80], as well as UV‐B exposure [55, 56], caused a reduced global DNA methylation level in plant cell cultures or intact plants. AAPH and high UV‐B exposure caused oxidative stress and increased plant tissue levels of NIC and the NIA metabolite trigonelline (TRIG; N‐methylnicotinic acid) (NIA itself was not analyzed) as well as increased defensive metabolism and gene expression [40, 54]. Interestingly, *Vicia cracca* L. plants, growing under chronic radiation exposure in the Chernobyl exclusion zone, contained increased levels of NIA and TRIG [81]. Although PARP activity was not measured, it is reasonable that AAPH, UV‐B, and radiation exposure activated PARP via increased levels of DNA strand breaks, leading to increased tissue levels of NIC and probably also NIA, which may be metabolized to TRIG. In addition, oxidative stress responses are common after exposure of plants to high concentrations of heavy metals [82], and heavy metal stress resulted in DNA hypomethylation [83]. In line with this, the DNA hypomethylating compounds NIC and NIA increased the tolerance of *Salix viminalis* clones against cadmium, copper, and zinc exposure [30] and the tolerance of *Pistia stratiotes* plants to cadmium stress was increased by NIC treatment [84]. Oxidative stress is also a common response to insect attack [85]. NIC treatment decreased pine weevil damage to Norway spruce seedlings and caused DNA hypomethylation in young seedlings [31]. Furthermore, redox‐active pollutants in the environment can give rise to epigenetic changes and transcriptional memory in plants as well as animals [86]. It is possible that oxidative stress caused by the abovementioned exposures would lead to elevated NIC/NIA levels, which in turn results in epigenetic effects and improved defense and tolerance.

The tripeptide glutathione (GSH) is, together with ascorbate in the ascorbate/glutathione cycle, essential for plant cell survival and involved in an array of cellular functions, such as detoxification, redox control, metabolism, and as antioxidant [87, 88]. GSH is also necessary for the biosynthesis of the heavy metal‐binding peptides phytochelatins [89]. Changes in the level and redox state of GSH can influence cell differentiation such as plant embryo development [90] and transportation of secondary metabolites such as anthocyanins into vacuoles [91]. The ratio between reduced and oxidized glutathione (GSH/GSSG) plays a decisive role for the redox state in the cell, in turn influencing epigenetic modifications and gene expression [92]. The redox state influences the access to some primary metabolites, such as methyl and acetyl groups provided from S‐adenosyl‐methionine (SAM) and acetyl‐CoA, respectively, needed for modification of DNA and histones [92, 93]. SAM also provides cysteine for the synthesis of GSH [94]. SAM synthetase seems to be regulated by the GSH/GSSG ratio [95]. Furthermore, in rats, glutathione transferase (GST) activity, important in detoxification processes, is stimulated by SAM [96]. Altogether, the connection between SAM and GSH is complex and needs deeper investigations.

In addition to DNA methylation, also the acetylation/deacetylation of histones, performed by the enzymes histone acetyltransferase (HAT) and histone deacetylase (HDAC), respectively, is redox‐regulated [92, 93].

Both NIC and NIA can cause a marked increase in cellular GSH in plant tissue [29]. There is a positive correlation between PARP activity and the increase in the GSH pool during growth in *Arabidopsis* cell culture [97]. Accordingly, PARP‐mediated NIC release, especially in connection to stress, may be a factor influencing GSH levels (Fig. 1).

GSH is especially important for the sensitivity of cells to specific plant growth factors and signaling molecules such as JA, SA, and indole acetic acid (IAA). The expression of genes associated with JA is connected to oxidative stress and influenced by GSH status, as reported by Han et al. [63]. These authors show that GSH status per se positively influences the expression of JA markers and JA responses and suggest that GSH is a key node for JA response, irrespective of the mechanism by which the GSH level is influenced. Thus, the NIC‐induced increase in GSH may be a factor promoting JA‐regulated defense. A similar GSH‐dependent growth factor response is seen regarding the stress signaling compounds SA [64] and IAA [98] pointing at the GSH level as a very important regulator for a general defense response by setting the level of sensitivity to specific signals.

The changes in stress signal/growth factor sensitivity fit well into observations that NIC promotes a strong increase in GSH in plant cell cultures as well as a general potentiation of defense [16] even though the mechanisms behind NIC‐induced changes of GSH level are not known. However, these effects caused by NIC might also be an effect of the NIC metabolite NIA, as we know that NIA can also increase GSH levels [29].

Accordingly, in a stress situation, increased GSH content as well as epigenetic changes, such as lowered DNA methylation and increased histone acetylation giving a more open chromatin, could allow stress signaling compounds present to induce defense gene expression (Fig. 1).

### Nicotinamidase and nicotinic acid

The enzyme nicotinamidase (EC:3.5.1.19), earlier annotated as isochorismatase in *Arabidopsis* [18], hydrolyzes NIC to NIA [59]. It is encoded in the genomes of various groups of organisms from bacteria to plants and invertebrates, but not in vertebrates. Nicotinamidase is an essential part of the NAD salvage pathway in plants, in which NIC is metabolized back to NAD [18]. Within the NAD salvage metabolism, nicotinamidase shows very high activity in duckweed when compared to other enzymes related to the metabolism and biosynthesis of NAD [99]. Nicotinamidase is via NIC closely associated with PARP activity. A nicotinamidase mutant in *A. thaliana* showed reduced PARP activity due to the block in NIC metabolization to NIA, causing increased NIC levels, leading to PARP inhibition [100]. The biological function of nicotinamidases in plants has mostly been studied for its direct role in the NAD salvage pathway, but research data also point at connections between ABA signaling and nicotinamidase activity [100, 101].

A role for NIA in defense activation has been suggested by Ahmad and coworkers [102], who showed that overexpression of *nicotinamidase 3* gene as well as exogenously applied NIA improved drought resistance in *Arabidopsis* plants.

Our findings since the original hypothesis suggest that NIA might be more active as a signaling compound after NIC metabolization rather than TRIG. Addition of NIA, but not TRIG, to plant cell cultures resulted in elevated GSH content, protection against cell leakage caused by the free radical generating compound AAPH, as well as increased activities of the TCA cycle enzymes aconitase and fumarase [29] and also DNA hypomethylation (unpublished Berglund and Ohlsson).

### PARP activity and NAD level

The involvement of active PARP [45] as well as downregulated PARP [46] in plant defense has been demonstrated. In both cases, NAD, essential for normal energetic metabolism in the cell [19], plays an important role.

Under nonstress conditions, the activity of PARP would be relatively low and the NAD level high enough to permit normal growth of the plant. Upon DNA damage, caused by, for example, oxidative stress, PARP activity will increase, leading to NAD degradation and formation of ADP‐ribose and NIC [35]. If PARP activity instead is decreased by synthetic inhibitors [45] or PARP knock‐out genes [46], the NAD level would elevate due to restricted degradation, which could lead to growth stimulation. However, if the NAD level becomes too high, it may be metabolized to cADPR and NIC. This has been demonstrated when the NAD pool is increased by inducible NAD overproduction, leading to the formation of cADPR, NIC, and NIA [103]. In fact, decreased PARP activity has been shown to stimulate ABA‐dependent stress signaling, which is suggested to be performed via cADPR formation from NAD [46]. One way for the production of cADPR and NIC by hydrolysis of NAD is mediated by the Toll and interleukin‐1 receptor‐like (TIR)‐based signaling against pathogens [104].

Obviously, it seems like extreme NAD levels, either too low or too high, dependent on high or low PARP activity, would result in increased levels of NIC, and probably also NIA. Increased levels of NIC and NIA could hypothetically indicate that the metabolism in the cells is seriously disturbed. This demands action: defense! Defense requires activation of gene expression. Many defense genes normally inactive due to epigenetic mechanisms (DNA methylation, histone modifications) may, if necessary, be prepared for activation by, for example, NIC and/or NIA.

Furthermore, if NIC or NIA is applied exogenously to the plant in non‐stressed conditions, the NAD supply could be kept at a reasonable level to sustain growth. First, since there is no stress situation, there is no need for high PARP activity, which would otherwise degrade NAD [37]; and second, NIC and NIA can help to keep a normal NAD level via the NAD salvage pathway [28]. At the same time, the levels of NIC and/or NIA would be high enough to open the door for a stress response, for example via epigenetic mechanisms. This would make it possible to achieve both normal growth and defense preparation.

### Some results from the literature in line with the presented hypothesis

Our results concerning the protective effects of NIC and NIA treatment of plants are supported by other investigations. NIC [105, 106, 107, 108, 109] and NIA [110] counteracted salinity stress. Plant quality and quantity were improved by spraying with NIC on plants grown on sandy soil, that is, in stress conditions [111]. Furthermore, drought tolerance was improved by exogenous application of NIA [102]. Protection against diseases caused by microorganisms was obtained by NIA [112] and by NIC [105, 113], while insect behavior was changed and insect mortality was increased for phloem‐feeding aphids by diets supplemented with NIC [114].

Increased contents of NIC and/or NIA in plant tissue under stress conditions have also been found, which can be interpreted as their formation playing a role as stress signaling compounds. Levels of these metabolites were increased in plants after exposure to *Plasmodiophora brassicae‐related* clubroot infection in *Brassica napus* [115], a *Trichoderma nigricans* strain that alleviated Cd‐induced oxidative stress [116], perfluorooctanesulfonic acid (PFOS) [117], radioactivity in the contaminated Chernobyl exclusion zone [81], and static magnetic field [118]. In *Pistia stratiotes* plants, NIC concentration was increased by Cd treatment at the same time as Cd toxicity was alleviated [84], and Pb toxicity in barley has been attenuated by NIC treatment [119]. Various growth parameters were improved by NIC in soybean [120] and other species reported by the same authors, in guar plants [121] and in lupine plants grown in sandy soil [122], and by NIA in barley plants [123].

Methods for application of NIC and NIA used in the above studies include seed treatment, foliar spray, and watering. The various reported improvements of NIC and NIA treatments indicate the mechanisms of these compounds to be of general nature among plant species.

## Conclusions and future perspectives

To summarize, we hypothesize that NIC and NIA, formed via PARP activated by oxidative stress and DNA damage, signal to the cell that defense is needed. The chromatin becomes more open through increased histone acetylation and decreased DNA methylation, giving access to the genome for appropriate stress signaling compounds present.

In other words, in contrast to treatment with a specific stress signaling compound, such as JA or SA, which are involved in their own respective response reactions [124], we propose that NIC and NIA would act in a more general manner, paving the way for subsequent action by the specific stress signaling compounds.

We suggest that, by these mechanisms, NIC and NIA could be used for the treatment of plants within agriculture, forestry, and horticulture, as sustainable, nontoxic, and inexpensive alternatives to the toxic insecticides, fungicides, etc., which are widely used nowadays. In addition, practical handling would be very simple if using seed treatment as the application method, even though watering and spraying the plants with the substances also should be feasible. We also see the possibility to utilize NIC/NIA‐producing microorganisms within agriculture and forestry to increase defense capacity and growth. This could, for example, be performed through the treatment of seeds with the microorganisms before sowing.

Hopefully, future plant research will further elucidate the defensive effects of NIC and NIA.

## Author contributions

TB and AO formulated the updated hypothesis and wrote the paper.

## Conflict of interest

The authors have no conflicts of interest to declare.

## Data Availability

Data sharing is not applicable to this article as no new data were created or analyzed in this study.
